# A Derivation Study of a Cardio-Nutrition-Inflammation-Oxygen Index and 3-Month Functional Outcomes After Outpatient Pulmonary Rehabilitation

**DOI:** 10.3390/nu18121879

**Published:** 2026-06-11

**Authors:** Sae Rom Kim, Jinkyeong Park, Ga Yang Shim, Seung Don Yoo, Eo Jin Park

**Affiliations:** 1 Department of Pulmonary, Allergy and Critical Care Medicine, Kyung Hee University Hospital at Gangdong, 892, Dongnam-ro, Gangdong-gu, Seoul 05278, Republic of Korea; 2 Department of Physical and Rehabilitation Medicine, Kyung Hee University College of Medicine, Kyung Hee University Hospital, 23, Kyungheedae-ro, Dongdaemun-gu, Seoul 02447, Republic of Korea; 3 Department of Rehabilitation Medicine, Kyung Hee University College of Medicine, Kyung Hee University Hospital at Gangdong, 892, Dongnam-ro, Gangdong-gu, Seoul 05278, Republic of Korea

**Keywords:** pulmonary rehabilitation, chronic respiratory disease, 6-min walk test, Short Physical Performance Battery, nutritional status

## Abstract

Background/Objectives: Short-term functional outcomes after outpatient pulmonary rehabilitation are heterogeneous. We examined whether a study-derived cardio-nutrition-inflammation-oxygen (CNIO) index integrating echocardiographic filling pressure, nutritional status, inflammation, and oxygen requirement was associated with 3-month functional outcomes in chronic respiratory disease. Methods: This single-center retrospective cohort study included 60 adults with chronic obstructive pulmonary disease, interstitial lung disease, or bronchiectasis who completed outpatient pulmonary rehabilitation and had baseline and 3-month functional assessments. The CNIO index was calculated as standardized E/e′ plus standardized ln(neutrophil-to-lymphocyte ratio) plus standardized resting oxygen flow rate minus standardized Geriatric Nutritional Risk Index, and the summed score was then standardized to mean 0 and SD 1. The primary outcome was 3-month 6 min walk test (6MWT) distance, and the exploratory secondary outcome was 3-month Short Physical Performance Battery (SPPB) score. The primary 6MWT analysis used multivariable analysis of covariance adjusted for baseline 6MWT, age, sex, body mass index, and diagnosis, whereas the exploratory SPPB analysis used ordinal logistic regression adjusted for baseline SPPB and the same covariates. Results: Mean 6MWT increased from 340.3 ± 61.0 m to 368.0 ± 102.0 m, corresponding to a mean change of 27.7 ± 90.3 m. Each 1-SD increase in CNIO was associated with a lower 3-month 6MWT distance (β = −43.42 m; 95% confidence interval [CI], −77.55 to −9.30; *p* = 0.014). In the exploratory ordinal logistic regression model for SPPB, each 1-SD increase in CNIO was associated with lower odds of being in a higher 3-month SPPB category, although the estimate was fragile and the confidence interval was close to the null (odds ratio = 0.39; 95% CI, 0.15 to 0.99; *p* = 0.048). Bootstrap internal stability analysis for the primary 6MWT model showed a wide percentile bootstrap 95% CI of −76.05 to −13.97 m per 1-SD increase in CNIO, supporting the need for cautious interpretation. Conclusions: In this hypothesis-generating derivation study, a higher standardized CNIO index was associated with lower 3-month 6MWT distance among adults with chronic respiratory disease who completed outpatient pulmonary rehabilitation. The association with SPPB was weaker and should be interpreted cautiously. These findings are not generalizable to patients who discontinue rehabilitation or are hospitalized for exacerbation during follow-up, and prospective external validation in larger, diagnostically stratified cohorts is required before CNIO can be considered for clinical risk stratification or rehabilitation planning.

## 1. Introduction

Pulmonary rehabilitation improves exercise capacity, symptoms, and health-related quality of life in patients with chronic respiratory diseases and is recommended as a core component of comprehensive respiratory care [1,2,3]. However, short-term functional response after rehabilitation is heterogeneous, and some patients show limited improvement despite completing a structured outpatient program [4,5]. Identifying baseline characteristics associated with less favorable follow-up performance is therefore clinically relevant for risk stratification and rehabilitation planning.

The 6 min walk test (6MWT) is widely used in pulmonary rehabilitation because it reflects integrated cardiopulmonary, muscular, and functional exercise performance [6,7]. The Short Physical Performance Battery (SPPB) provides complementary information on lower-extremity function through balance, gait speed, and chair-rise performance [8,9]. Together, these measures capture related but distinct aspects of functional status after rehabilitation, and their responsiveness may differ across patient populations and outcome domains [5,7,10].

Although several clinical and physiologic factors have been associated with rehabilitation outcomes, prediction of functional response remains challenging [4,11]. Baseline respiratory impairment alone may not fully explain rehabilitation-related outcome variability, particularly in chronic respiratory diseases with systemic manifestations [4,12,13]. Previous studies have highlighted the relevance of multidimensional clinical profiling, physical performance, nutritional status, inflammatory burden, and comorbidity-related factors, but routinely available multidomain indices for short-term functional outcomes after outpatient pulmonary rehabilitation remain insufficiently established.

Cardiac filling pressure, nutritional risk, systemic inflammation, and oxygen requirement may represent clinically relevant but different aspects of baseline vulnerability [14,15,16,17]. Higher E/e′ may reflect impaired filling reserve, lower Geriatric Nutritional Risk Index may indicate poorer nutritional and muscle reserve, higher neutrophil-to-lymphocyte ratio may reflect systemic inflammatory burden, and higher resting oxygen requirement may indicate greater cardiopulmonary limitation at rehabilitation entry [17,18,19,20]. In routine practice, these factors are often considered separately rather than as a unified baseline profile.

Against this background, the present study was designed as a retrospective derivation study to examine whether a study-derived cardio-nutrition-inflammation-oxygen index integrating E/e′, Geriatric Nutritional Risk Index, ln(neutrophil-to-lymphocyte ratio), and resting oxygen requirement was associated with 3-month functional outcomes after outpatient pulmonary rehabilitation in a mixed cohort of chronic obstructive pulmonary disease, interstitial lung disease, and bronchiectasis. We hypothesized that a higher, less favorable CNIO score would be independently associated with lower 3-month 6MWT distance after adjustment for baseline function and key clinical covariates, and we additionally explored whether a similar association was observed for 3-month SPPB performance.

## 2. Methods

### 2.1. Study Design

This single-center retrospective cohort study used data from an outpatient pulmonary rehabilitation program at Kyung Hee University Hospital at Gangdong. Baseline pulmonary rehabilitation assessments were conducted between March 2022 and October 2025, and 3-month outcome assessments were obtained within a predefined follow-up window of 90 ± 14 days. The last routine-care follow-up assessment included in the retrospective dataset was completed in February 2026, and data extraction and analysis for the present study were performed after institutional review board approval.

### 2.2. Participants

Adults aged 19 years or older with bronchiectasis, interstitial lung disease, or chronic obstructive pulmonary disease who were enrolled in twice-weekly outpatient pulmonary rehabilitation were screened for eligibility. Inclusion required completion of the planned program, baseline and follow-up 6MWT and SPPB data within 90 ± 14 days after baseline, echocardiographic E/e′ within 1 month of baseline, laboratory data for GNRI and NLR calculation within 1 month of baseline, and a recorded baseline resting oxygen flow rate.

Patients were excluded for acute coronary syndrome or acute decompensated heart failure during the first three months of rehabilitation, hospitalization for acute respiratory exacerbation during follow-up, severe orthopedic or neurologic gait impairment preventing 6MWT completion, or missing key exposure or outcome data. Because patients who discontinued rehabilitation or experienced exacerbation-related hospitalization were excluded by design, the findings apply only to a selected subgroup of rehabilitation completers without major intercurrent events.

During the study period, 108 patients were referred to or clinically considered for outpatient pulmonary rehabilitation. Of these, 36 patients were not screened for study eligibility, including 20 who were not referred after initial clinical consideration and 16 who declined rehabilitation or did not attend the initial pulmonary rehabilitation assessment. The remaining 72 patients were screened for study eligibility after pulmonary rehabilitation referral or assessment. Twelve patients were excluded after screening, including 7 who did not complete the planned outpatient pulmonary rehabilitation program, 2 who had unavailable echocardiographic E/e′ data within the prespecified baseline window, 1 who had unavailable laboratory data required for GNRI and NLR calculation, 1 who had unavailable baseline or 3-month functional outcome data within the prespecified assessment window, and 1 who was hospitalized for acute respiratory exacerbation during follow-up. The final analytic cohort included 60 patients. Accordingly, the analytic cohort represents a selected subgroup of patients who were referred to or assessed for outpatient pulmonary rehabilitation, completed the planned program, and had complete baseline and follow-up data.

**Figure 1:**
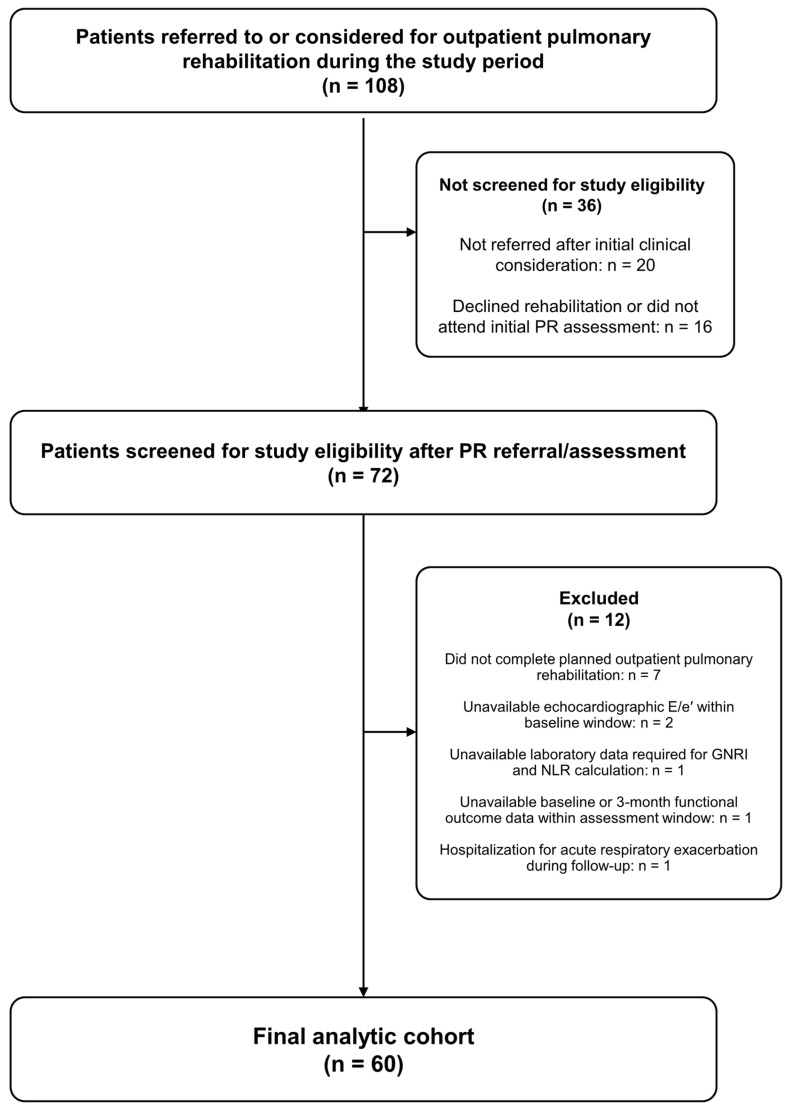
Flow diagram of cohort assembly and reasons for exclusion. E/e′ = ratio of early mitral inflow velocity to early diastolic mitral annular velocity; GNRI = Geriatric Nutritional Risk Index; NLR = neutrophil-to-lymphocyte ratio.

A total of 108 patients were referred to or clinically considered for outpatient pulmonary rehabilitation during the study period. Thirty-six patients were not screened for study eligibility, including 20 who were not referred after initial clinical consideration and 16 who declined rehabilitation or did not attend the initial pulmonary rehabilitation assessment. Seventy-two patients were screened for study eligibility after pulmonary rehabilitation referral or assessment. Twelve patients were excluded after screening, including 7 who did not complete the planned outpatient pulmonary rehabilitation program, 2 who lacked echocardiographic E/e′ data within the predefined baseline window, 1 who lacked laboratory data required for GNRI and NLR calculation, 1 who lacked baseline or 3-month functional outcome data within the predefined assessment window, and 1 who was hospitalized for acute respiratory exacerbation during follow-up. The final analytic cohort included 60 patients. The cohort assembly process and reasons for exclusion are shown in Figure 1.

The study protocol was reviewed and approved by the Institutional Review Board of Kyung Hee University Hospital at Gangdong (IRB No. 2026-03-039). The requirement for informed consent was waived due to the retrospective nature of the study, in accordance with applicable institutional requirements and the Declaration of Helsinki.

### 2.3. Data Collection and Variables

Clinical data were retrospectively obtained from electronic medical records and pulmonary rehabilitation assessment records. The baseline demographic and anthropometric variables included age, sex, body mass index (BMI), and diagnosis category (COPD, ILD, or bronchiectasis). Age was recorded in years, and BMI was recorded in kg/m^2^.

The outpatient pulmonary rehabilitation program consisted of supervised twice-weekly sessions for 12 weeks. It included breathing retraining, aerobic exercise, resistance training for major muscle groups, and education on energy conservation and symptom management [1,2]. Exercise prescriptions were individualized according to baseline function and clinical status and were adjusted during follow-up.

Four baseline domains, echocardiographic filling pressure, nutritional status, inflammatory burden, and oxygen requirement, deemed clinically relevant to functional status at pulmonary rehabilitation admission were included in the study [9,19,21]. The echocardiographic variable of interest, E/e′, was derived from transthoracic echocardiography conducted within one month of the baseline pulmonary rehabilitation assessment [14,18]. GNRI and NLR were calculated using laboratory data collected within one month of baseline. GNRI was calculated using a standard published formula based on serum albumin, actual body weight, and ideal body weight, and NLR was calculated as the absolute neutrophil count divided by the absolute lymphocyte count [19,21]. Because NLR was expected to have a right-skewed distribution, a natural logarithmic transformation was applied, and ln(NLR) was used in the regression and composite score analyses. Resting oxygen requirement was defined as the prescribed oxygen flow rate recorded at rest at the baseline assessment and was analyzed as a continuous variable in L/min [17].

To assess whether the association between CNIO and 3-month outcomes persisted after adjustment for baseline lung function, FEV1 % predicted was included in planned sensitivity analyses. Because the cohort included COPD, ILD, and bronchiectasis, this was intended as a pragmatic adjustment for spirometric status rather than a diagnosis-equivalent measure of disease severity across all groups.

### 2.4. Outcome Measures

The primary outcome was 3-month 6MWT distance in meters. Baseline 6MWT was also recorded, and the change from baseline was summarized descriptively.

The exploratory secondary outcome was 3-month SPPB score on the conventional 0 to 12 scale [8,9]. Baseline SPPB was also recorded, and the change from baseline was summarized descriptively.

With a window of 90 ± 14 days, the follow-up outcome assessment was scheduled to take place approximately three months after baseline. An analysis of covariance (ANCOVA) framework was used to model 3-month outcome values in the primary analyses while accounting for relevant baseline values.

### 2.5. Exposure/Predictor Measures

The study-derived CNIO index, which combines four routinely available baseline domains into a single composite indicating a less favorable functional profile at pulmonary rehabilitation admission, was the primary exposure of interest. The CNIO index was first calculated as the sum of four cohort-standardized baseline components: z(E/e′) + z(ln[NLR]) + z(resting oxygen flow rate) + z(−GNRI). The negative GNRI term was used so that lower nutritional status contributed to a higher, less favorable CNIO value. In the present cohort, the means and standard deviations used for standardization were 11.65 ± 2.71 for E/e′, 0.78 ± 0.49 for ln(NLR), 2.01 ± 0.93 L/min for resting oxygen flow rate, and 99.03 ± 6.24 for GNRI. Thus, the unstandardized summed composite can be expressed as [(E/e′ − 11.65)/2.71] + [(ln[NLR] − 0.78)/0.49] + [(resting O_2_ L/min − 2.01)/0.93] − [(GNRI − 99.03)/6.24]. The observed SD of this summed composite was 2.53 in the analytic cohort; therefore, the summed composite was divided by 2.53 for the regression analyses so that the reported CNIO coefficient represented the association per 1-SD increase in this cohort-specific composite. This cohort-specific standardization limits direct transportability to other populations unless the same standardization parameters are applied. The equal-weighted formulation was chosen on clinical grounds to create a transparent derivation construct and was not statistically optimized for outcome prediction; other weighting schemes might yield different results. Accordingly, CNIO should be interpreted as a provisional, study-derived exposure rather than as a validated prognostic tool.

The four CNIO components, E/e′, GNRI, ln(NLR), and resting oxygen flow rate, were concurrently included in pre-specified interpretability models in addition to the composite index analysis to investigate the relationship between each component and the 3-month outcomes following adjustment for the same clinical covariates used in the primary models. Because the cohort size was modest relative to the number of simultaneously modeled components and covariates, these component-level analyses were interpreted as exploratory and were not intended to support definitive inference regarding the independent effect of any single component.

### 2.6. Statistical Analysis

Baseline characteristics and functional outcomes were summarized descriptively. Categorical variables were presented as counts and percentages, and continuous variables were presented as mean ± standard deviation or median (interquartile range), as appropriate.

Using multivariable ANCOVA, the main analysis examined the relationship between the CNIO index and the 3-month 6MWT. The primary model is specified as follows:

6MWT_3mo ~ CNIO + 6MWT_baseline + age + sex + BMI + dx

The exploratory secondary analysis examined 3-month SPPB using ordinal logistic regression because SPPB is a bounded ordinal scale. The model included CNIO, baseline SPPB, age, sex, BMI, and diagnosis.

SPPB_3mo category ~ CNIO + SPPB_baseline + age + sex + BMI + dx

The proportional-odds coefficient was exponentiated and presented as an odds ratio for being in a higher 3-month SPPB category. The proportional-odds assumption was evaluated using threshold-specific binary logistic regression diagnostics as a Brant-type assessment; because some SPPB thresholds were sparse, this diagnostic was interpreted descriptively rather than as a definitive model-selection test.

The reference group for diagnosis was bronchiectasis, and diagnosis category was added as a categorical covariate. In the 6MWT ANCOVA model, the regression coefficient for CNIO was interpreted as the change in 3-month 6MWT distance per 1-SD increase in the standardized CNIO index. In the SPPB ordinal logistic regression model, the exponentiated CNIO coefficient was interpreted as the proportional odds ratio for being in a higher 3-month SPPB category per 1-SD increase in the standardized CNIO index. *p*-values, 95% confidence intervals (CIs), and model fit indices were presented as appropriate for each model.

Planned component models were fitted for each outcome by simultaneously entering the four CNIO components rather than the composite score to enhance interpretability. For the 6MWT, the component model was as follows:

6MWT_3mo ~ E/e′ + GNRI + ln(NLR) + resting O_2_ L/min + 6MWT_baseline + age + sex + BMI + dx

For SPPB, the component model was as follows:

SPPB_3mo ~ E/e′ + GNRI + ln(NLR) + resting O_2_ L/min + SPPB_baseline + age + sex + BMI + dx

The multivariable ANCOVA model for 3-month 6MWT was defined as the primary analysis, and the ordinal logistic regression model for 3-month SPPB was defined as an exploratory secondary analysis. Linear ANCOVA for SPPB, component models, FEV1-adjusted models, and leave-one-diagnosis-out analyses were intended as supportive exploratory or sensitivity analyses to assess interpretability and pattern consistency rather than to support definitive inference regarding individual components or diagnosis-specific heterogeneity. Because these exploratory analyses were intended to assess pattern consistency rather than to provide confirmatory inference, no formal multiplicity correction was applied; therefore, *p*-values from component models and sensitivity analyses were interpreted as descriptive only, with emphasis placed on effect-size direction, confidence intervals, and consistency with the main analysis.

Because this was a retrospective derivation study including all consecutively eligible patients, no formal a priori sample-size calculation was performed. For the primary 6MWT model, 60 participants were analyzed with seven modeled predictors, corresponding to approximately 8.6 observations per predictor. The modest sample size increased the risk of model instability and overfitting; therefore, the analyses were interpreted with emphasis on effect-size direction, confidence intervals, and internal stability rather than on definitive prediction performance.

To examine internal stability and possible overfitting in the primary 6MWT model, bootstrap resampling with 1000 resamples was performed for the CNIO coefficient and model R^2^, and percentile bootstrap confidence intervals were reported. Multicollinearity was assessed using variance inflation factors. Because SPPB is a bounded ordinal scale, ordinal logistic regression was specified as an exploratory secondary analysis for 3-month SPPB, while the linear ANCOVA model for SPPB was retained as a sensitivity analysis.

No imputation was performed, and all analyses were conducted in the complete-case analytic cohort. All statistical tests were two-sided. A *p*-value < 0.05 was considered statistically significant for the primary 6MWT analysis, whereas *p*-values from exploratory secondary, component, and sensitivity analyses were interpreted descriptively. Analyses were performed using R software (version 4.2.3; R Foundation for Statistical Computing, Vienna, Austria), including the stats package for linear models, the car package for variance inflation factors, the MASS package for ordinal logistic regression, and the boot package for bootstrap resampling.

## 3. Results

### 3.1. Baseline Characteristics

Sixty patients were included in the final analysis. Table 1 shows baseline characteristics overall and by diagnosis category. Descriptively, patients with ILD tended to show a less favorable baseline profile than those with COPD or bronchiectasis, including higher E/e′, higher resting oxygen requirement, and lower baseline functional measures. Mean age was 63.8 ± 9.0 years, 68.3% were male, and mean BMI was 21.4 ± 2.7 kg/m^2^. Diagnoses included COPD in 41.7%, ILD in 33.3%, and bronchiectasis in 25.0%. Mean baseline E/e′ was 11.7 ± 2.7, GNRI was 99.0 ± 6.2, NLR was 2.5 ± 1.3, resting oxygen requirement was 2.0 ± 0.9 L/min, and FEV1 % predicted was 81.7 ± 10.7. Mean baseline 6MWT distance was 340.3 ± 61.0 m, mean baseline SPPB was 8.7 ± 1.5, and mean standardized CNIO index was −0.00 ± 1.00. Because CNIO was standardized in the full analytic cohort, diagnosis-specific CNIO means represent deviations from the overall cohort average, with positive values indicating a less favorable baseline profile. The initial summed CNIO composite before final standardization had an observed SD of 2.53. Correlations among the four CNIO components were modest, with absolute pairwise correlations ranging from 0.06 to 0.29 (Supplementary Table S5). These modest correlations suggest that the four components captured related but non-redundant baseline domains, although they do not establish that equal weighting is optimal.

### 3.2. Functional Outcomes at 3 Months

At 3 months, mean 6MWT distance was 368.0 ± 102.0 m compared with 340.3 ± 61.0 m at baseline, corresponding descriptively to a mean change of 27.7 ± 90.3 m. The variability in 6MWT change was large, with the standard deviation of change exceeding the mean change. In contrast, mean SPPB was 8.6 ± 2.9 points at 3 months versus 8.7 ± 1.5 points at baseline, corresponding descriptively to a mean change of −0.08 ± 2.57 points (Table 2). Individual change was heterogeneous. Twenty-eight patients (46.7%) achieved a 6MWT improvement of at least 30 m, whereas 25 patients (41.7%) had a lower 6MWT distance at 3 months than at baseline. For SPPB, 27 patients (45.0%) improved, 7 patients (11.7%) were unchanged, and 26 patients (43.3%) worsened. The mean 6MWT improvement of 27.7 m was close to but below the commonly cited 25–35 m minimal important difference range, indicating that group-level functional improvement should be interpreted cautiously [22,23,24].

### 3.3. Primary Analysis: 3-Month 6MWT

In the primary ANCOVA model, each 1-SD increase in the standardized CNIO index was associated with a lower 3-month 6MWT distance after multivariable adjustment for baseline 6MWT, age, sex, BMI, and diagnosis (β = −43.42 m; 95% CI, −77.55 to −9.30; *p* = 0.014). Baseline 6MWT was positively associated with 3-month 6MWT, and ILD was associated with a lower 3-month 6MWT relative to bronchiectasis. The adjusted R^2^ was 0.509 (Table 3). To examine whether the CNIO estimate was materially dependent on adjustment for diagnosis, we fitted an otherwise identical sensitivity model without diagnosis. In this model, each 1-SD increase in CNIO remained associated with lower 3-month 6MWT distance (β = −58.85 m; 95% CI, −81.39 to −36.32; *p* < 0.001; adjusted R^2^ = 0.484).

### 3.4. Exploratory Secondary Analysis: 3-Month SPPB

In the exploratory ordinal logistic regression model, each 1-SD increase in the standardized CNIO index was associated with lower odds of being in a higher 3-month SPPB category after adjustment for baseline SPPB, age, sex, BMI, and diagnosis (odds ratio = 0.39; 95% CI, 0.15 to 0.99; *p* = 0.048). Baseline SPPB was positively associated with higher follow-up SPPB category, whereas diagnosis category was not. Because the confidence interval was wide and close to the null, and because group-level SPPB change was minimal, this finding was interpreted as exploratory and not robust. Threshold-specific Brant-type diagnostics did not show statistically significant non-proportionality for the CNIO term (*p* = 0.172), but this diagnostic was interpreted descriptively because some SPPB thresholds were sparse. The linear ANCOVA sensitivity analysis showed a consistent inverse association (β = −1.07 points per 1-SD increase in CNIO; 95% CI, −2.11 to −0.02; *p* = 0.045; Supplementary Table S1). Multicollinearity diagnostics did not suggest severe collinearity. The VIF for CNIO was 3.34 in the 6MWT model and 3.30 in the SPPB model. In the exploratory component models, the maximum VIF was 2.48 for the 6MWT model and 2.46 for the SPPB model. Bootstrap internal stability analysis of the primary 6MWT model showed a percentile bootstrap 95% CI of −76.05 to −13.97 m for the CNIO coefficient per 1-SD increase. The apparent R^2^ was 0.567, and the optimism-corrected R^2^ was 0.457.

### 3.5. Exploratory Component Models

Detailed component-model results are provided only in Supplementary Table S2. Due to the modest sample size, simultaneous inclusion of multiple related CNIO components and clinical covariates, and potential collinearity, individual component coefficients were considered unstable and should not be interpreted as evidence for or against any specific mechanism. These models were retained only as exploratory interpretability analyses and were not used to infer the independent contribution of any single CNIO component.

### 3.6. Sensitivity Analyses

**Figure 2:**
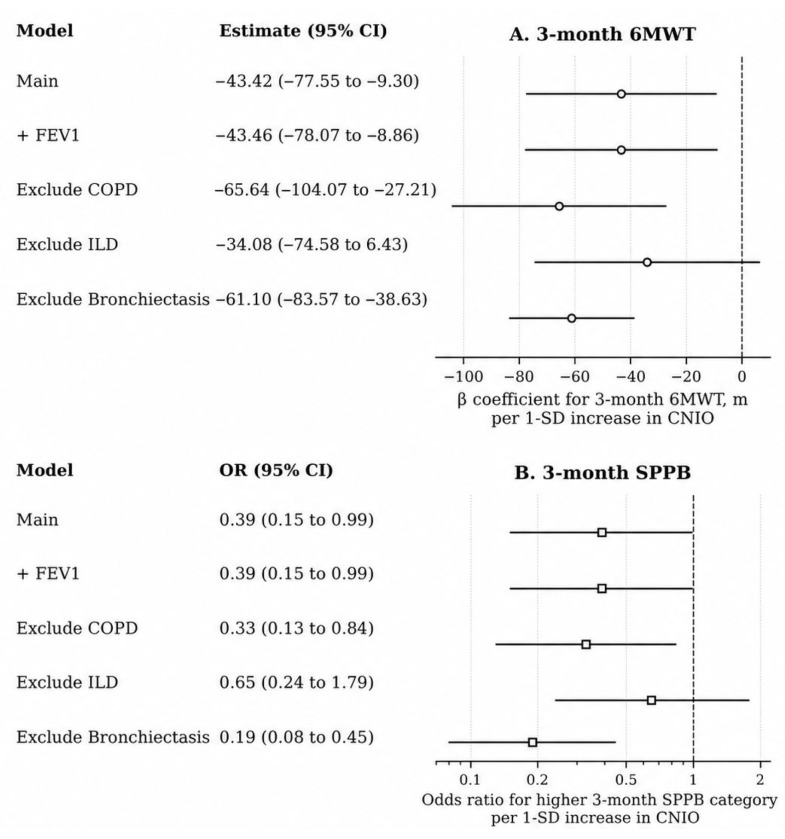
Forest plots of CNIO coefficients in the main and sensitivity analyses.

After additional adjustment for FEV1 % predicted, the association between standardized CNIO and 3-month 6MWT remained significant (β = −43.46 m; 95% CI, −78.07 to −8.86; *p* = 0.015; Supplementary Table S3). In the FEV1-adjusted ordinal logistic regression model for SPPB, each 1-SD increase in CNIO remained associated with lower odds of being in a higher 3-month SPPB category (odds ratio = 0.39; 95% CI, 0.15 to 0.99; *p* = 0.047; Supplementary Table S1). However, FEV1 % predicted was interpreted as a pragmatic spirometric adjustment rather than as an equivalent severity measure across COPD, ILD, and bronchiectasis. The CNIO estimates from the main and sensitivity analyses are summarized in Figure 2.

Regression coefficients for 3-month 6MWT and proportional odds ratios for 3-month SPPB are shown for the standardized CNIO index. The 6MWT models report β coefficients in meters per 1-SD increase in CNIO. The SPPB ordinal logistic regression models report odds ratios for being in a higher 3-month SPPB category per 1-SD increase in CNIO. The main models were adjusted for the baseline value of the corresponding outcome, age, sex, BMI, and diagnosis. The FEV1-adjusted models included the same covariates plus FEV1 (% predicted). Leave-one-diagnosis-out models were adjusted for the baseline value of the corresponding outcome, age, sex, and BMI. Horizontal bars indicate 95% confidence intervals. Negative β values for 6MWT and odds ratios below 1 for SPPB indicate poorer 3-month outcomes with higher CNIO. The SPPB estimates are exploratory and should be interpreted cautiously because confidence intervals were wide and close to the null in the main model.

### 3.7. Exploratory Diagnosis-Specific Analyses

Leave-one-diagnosis-out sensitivity analyses are reported in Supplementary Table S4. The inverse association between CNIO and both 3-month 6MWT and 3-month SPPB was attenuated after exclusion of patients with ILD, suggesting that the overall signal may have been influenced in part by the ILD subgroup. This finding raises the possibility that CNIO may partly reflect ILD-related disease burden rather than a fully generalizable multidomain vulnerability index across all diagnostic groups. Given the reduced sample size within each diagnostic subset, these findings should be interpreted only as exploratory pattern-consistency analyses rather than definitive evidence of diagnosis-specific heterogeneity.

## 4. Discussion

In this single-center retrospective derivation cohort, a higher standardized CNIO index was associated with lower 3-month functional outcomes after outpatient pulmonary rehabilitation, with a more consistent association for 6MWT than for SPPB. The primary finding was that a less favorable multidomain baseline profile remained associated with lower follow-up walking capacity after adjustment for baseline performance and selected clinical covariates. The exploratory SPPB finding, evaluated using ordinal logistic regression, was directionally consistent but weaker and should be interpreted more cautiously. Because the CNIO construct was study-derived and evaluated in the same cohort, these findings should be interpreted as hypothesis-generating rather than as validation of a clinical prediction tool.

The association with 6MWT is consistent with previous observations that response to pulmonary rehabilitation is heterogeneous and is not fully explained by spirometric impairment alone. Prior studies have emphasized the importance of multidimensional clinical profiling, physical performance measures, nutritional status, and systemic inflammation in chronic respiratory disease. The present study extends this concept by examining a simple study-derived composite that integrates cardiac filling pressure, nutritional risk, inflammatory burden, and oxygen requirement. However, unlike validated prognostic models, CNIO was derived and tested within the same cohort, and therefore its apparent association with follow-up function should be considered preliminary.

The 6MWT finding is clinically plausible because the CNIO construct integrates domains that may collectively reflect physiologic vulnerability at rehabilitation entry [14,17,25]. Higher E/e′ may indicate reduced filling reserve, lower GNRI may reflect poorer nutritional and muscle reserve, higher ln(NLR) may reflect inflammatory burden, and greater resting oxygen requirement may indicate more advanced cardiopulmonary limitation [14,17,19,25]. The pathophysiological interpretation of CNIO should be cautious. Although the combination of cardiac filling pressure, nutritional status, inflammatory burden, and oxygen requirement is biologically plausible, the present data do not demonstrate that these domains act synergistically or causally in determining rehabilitation outcomes. Rather, CNIO may function mainly as a marker of overall disease burden or global physiologic vulnerability rather than as evidence of a specific multidomain mechanism associated with rehabilitation response. This interpretation is supported by the attenuation of the association after excluding patients with ILD and by the persistence of a diagnosis effect for ILD in the 6MWT model. This possibility is particularly relevant because the ILD subgroup had a less favorable baseline profile and because FEV1 % predicted may not capture disease burden equivalently across COPD, ILD, and bronchiectasis. The diagnosis coefficient for ILD also deserves comment. ILD remained associated with lower 3-month 6MWT distance after adjustment for CNIO and other clinical covariates, suggesting that CNIO did not fully explain diagnosis-related differences in follow-up walking capacity. Residual disease-specific factors not captured by CNIO, such as diffusion limitation, exertional desaturation, radiologic extent, or fibrosis-related progression, may have contributed to this association. In exploratory, underpowered component models, GNRI showed the most consistent individual association with 3-month 6MWT. However, this finding is not robust and should not be interpreted as evidence that GNRI is the dominant driver of the composite association, because the component model included multiple related domains and clinical covariates in a modest sample. In this setting, null findings for the remaining components may reflect collinearity and model instability as much as true absence of contribution. However, because the CNIO index was specified and evaluated within the same cohort, the present findings should not be interpreted as evidence that this weighting scheme is optimal or transportable to other clinical populations.

The exploratory secondary SPPB analysis was less robust than the 6MWT analysis. Although the ordinal logistic regression model showed that higher CNIO was associated with lower odds of being in a higher 3-month SPPB category, the confidence interval was close to the null, and group-level SPPB change was minimal over follow-up. The linear ANCOVA sensitivity analysis showed a consistent inverse direction, but it should be interpreted cautiously because SPPB is a bounded ordinal scale. Together, these results suggest that SPPB may have been less responsive than 6MWT for detecting short-term functional variation in this outpatient rehabilitation cohort [5,10]. The mean 6MWT change deserves clinical caution. Although the direction of change suggested improvement, the group-level mean increase of 27.7 m was close to but below the commonly cited 25–35 m minimal important difference range, and individual responses were heterogeneous. Therefore, the present findings should be interpreted as associations with follow-up functional status rather than as evidence of clinically meaningful improvement for the cohort as a whole.

Sensitivity analyses suggested that the observed associations were not fully uniform across diagnostic subsets. In particular, the CNIO association with both 3-month 6MWT and 3-month SPPB was attenuated after exclusion of ILD, which raises the possibility that the overall signal may have been influenced in part by the ILD subgroup. However, these reduced models involved substantially smaller sample sizes and wider uncertainty, so the findings should not be interpreted as evidence of true diagnosis-specific effect modification. In addition, the FEV1 % predicted sensitivity analysis should be interpreted cautiously because spirometric severity may not reflect disease burden equivalently across COPD, ILD, and bronchiectasis. These subset findings are therefore best regarded as exploratory. The potential clinical utility of CNIO remains provisional. At present, CNIO should not be used to guide rehabilitation prescriptions, clinical treatment decisions, or patient selection. If externally validated in larger prospective cohorts, a refined version of CNIO might eventually help identify patients who require closer monitoring during pulmonary rehabilitation. However, such use would require validation in diagnostically stratified cohorts and assessment of whether CNIO adds prognostic information beyond disease-specific severity measures. Selection of rehabilitation completers is an important limitation. By restricting the analytic cohort to patients who completed the planned rehabilitation program and had complete follow-up data, the study may have selected a healthier and more adherent subgroup than the broader real-world pulmonary rehabilitation population. Therefore, the findings should not be generalized to patients who discontinue rehabilitation, experience exacerbation-related hospitalization, or lack complete baseline and follow-up testing.

This study has several strengths, including clinically relevant outcomes, a structured outpatient pulmonary rehabilitation setting, adjustment for baseline outcome values, and multiple sensitivity analyses. However, several limitations should be considered. The retrospective single-center design limits causal inference and generalizability. The study period overlapped with the post-acute phase of the COVID-19 pandemic, and changes in rehabilitation delivery, referral patterns, infection-related morbidity, or patient adherence during this period may have influenced outcomes. The modest sample size increases the possibility of model instability, particularly in the component models and diagnosis-specific subset analyses, where the number of estimated parameters was high relative to the available sample size. Although bootstrap resampling was used to examine internal stability of the primary 6MWT model, no independent internal split-sample validation or external validation cohort was available. In addition, the study was not powered a priori, and the modest sample size limited the reliability of exploratory component-level, ordinal, and diagnosis-subset analyses. Selection bias is a major limitation because the analytic cohort was restricted to patients who completed the planned outpatient rehabilitation program and had complete baseline and follow-up data. The findings may therefore not be generalizable to patients who discontinue rehabilitation early, experience exacerbation-related hospitalization, have greater clinical instability, or lack complete baseline testing. This completer-based design may also introduce immortal time or survivor bias because patients had to remain alive, clinically stable, and assessable through the 3-month follow-up window. Residual confounding by comorbidity burden, medication changes, corticosteroid exposure, nutritional interventions, oxygen-prescription changes, and rehabilitation adherence remains possible. Medication use, including beta-blockers and systemic or inhaled corticosteroids, may have influenced exercise tolerance or inflammatory markers such as NLR, but these factors could not be fully incorporated into the models. E/e′ is a noninvasive surrogate of left ventricular filling pressure rather than a direct invasive measurement and may be less reliable in certain clinical settings, such as atrial fibrillation or significant mitral valve disease, which were not fully characterized in this dataset. Inter-rater or inter-reader reliability for echocardiographic E/e′ measurement was not assessed. Adjustment for FEV1 % predicted also has limitations because FEV1 is not an equivalent severity measure across COPD, ILD, and bronchiectasis; DLCO or ILD-specific indices such as the GAP index may have been more informative but were not consistently available. Finally, the CNIO index was study-derived and evaluated in the same cohort in which it was specified; accordingly, the present findings should be viewed as associative and hypothesis-generating rather than as validation of a clinical prediction tool. External validation in an independent cohort will be necessary before any broader prognostic or stratification use can be considered.

## 5. Conclusions

In this hypothesis-generating derivation study, a higher standardized CNIO index was associated with lower 3-month 6MWT distance among adults with chronic respiratory disease who completed outpatient pulmonary rehabilitation. The association with SPPB, evaluated using an ordinal model, was weaker and remains provisional. These findings are not generalizable to patients who discontinue rehabilitation or experience exacerbation-related hospitalization during follow-up. Prospective external validation in larger and diagnostically stratified cohorts is required before CNIO can be considered for clinical risk stratification or rehabilitation planning.

## Figures and Tables

**Table 1:** Baseline Characteristics Overall and by Diagnosis Category.

Characteristic	Overall (*N* = 60)	COPD (*n* = 25)	ILD (*n* = 20)	Bronchiectasis (*n* = 15)
Age, years	63.8 ± 9.0	60.9 ± 8.6	64.7 ± 9.2	67.6 ± 8.3
Male, *n* (%)	41 (68.3)	16 (64.0)	15 (75.0)	10 (66.7)
Female, *n* (%)	19 (31.7)	9 (36.0)	5 (25.0)	5 (33.3)
BMI, kg/m^2^	21.4 ± 2.7	21.6 ± 2.6	21.2 ± 3.1	21.3 ± 2.6
E/e′	11.7 ± 2.7	9.9 ± 2.1	13.7 ± 2.3	11.9 ± 2.3
GNRI	99.0 ± 6.2	101.9 ± 5.6	97.3 ± 4.6	96.6 ± 7.5
NLR	2.5 ± 1.3	2.0 ± 0.9	3.2 ± 1.7	2.2 ± 0.8
Resting oxygen requirement, L/min	2.0 ± 0.9	1.4 ± 0.7	2.6 ± 0.9	2.2 ± 0.6
FEV1 (% predicted)	81.7 ± 10.7	87.1 ± 10.4	77.7 ± 9.8	78.3 ± 8.7
6MWT at baseline, m	340.3 ± 61.0	359.3 ± 59.7	315.4 ± 65.6	342.1 ± 47.0
SPPB at baseline, points	8.7 ± 1.5	9.4 ± 1.3	8.0 ± 1.6	8.5 ± 1.4
CNIO index	−0.00 ± 1.00	−0.83 ± 0.67	0.87 ± 0.63	0.20 ± 0.75

Data are presented as mean ± standard deviation or *n* (%). The CNIO index was standardized to mean 0 and SD 1 in the full analytic cohort; positive values indicate a less favorable profile than the cohort average. The CNIO index shown is the final standardized composite used in the regression models; before final standardization, the summed CNIO composite had an observed SD of 2.53. NLR, ln(NLR), and resting oxygen requirement were also summarized as median (interquartile range): NLR, 2.0 (1.5–3.2); ln(NLR), 0.70 (0.39–1.17); and resting oxygen requirement, 2.0 (1.5–2.5) L/min. CNIO = cardio-nutrition-inflammation-oxygen; BMI = body mass index; ILD = interstitial lung disease; GNRI = Geriatric Nutritional Risk Index; NLR = neutrophil-to-lymphocyte ratio; FEV1 = forced expiratory volume in 1 s; 6MWT = 6 min walk test; SPPB = Short Physical Performance Battery. Baseline distributions across diagnosis categories are presented descriptively; no hypothesis tests were performed for between-diagnosis comparisons.

**Table 2:** Outcomes at Baseline and 3 months.

Outcome	Baseline	3 Months	Change
6MWT, m	340.3 ± 61.0	368.0 ± 102.0	27.7 ± 90.3
SPPB, points	8.7 ± 1.5	8.6 ± 2.9	−0.08 ± 2.57

Data are presented as mean ± standard deviation. Change was calculated as the 3-month value minus the baseline value. 6MWT = 6 min walk test; SPPB = Short Physical Performance Battery. Data are presented descriptively as mean ± standard deviation; no paired hypothesis tests were performed for within-cohort change.

**Table 3:** Primary ANCOVA Model for 6MWT and Exploratory Ordinal Logistic Regression Model for SPPB at 3 Months. (**A**) 6MWT at 3 months (Primary Analysis). (**B**) SPPB at 3 Months (Exploratory Ordinal Logistic Regression Analysis).

**(A)**			
**Variable**	**β**	**95% CI**	***p*** **-Value**
CNIO index	−43.42	−77.55 to −9.30	0.014
6MWT at baseline, m	0.48	0.11 to 0.86	0.013
Age, years	−0.66	−3.30 to 1.97	0.617
Male sex	−1.01	−45.07 to 43.06	0.964
BMI, kg/m^2^	1.39	−6.10 to 8.88	0.711
COPD	−19.60	−75.42 to 36.22	0.484
ILD	−62.20	−119.85 to −4.54	0.035
**(B)**			
**Variable**	**OR**	**95% CI**	***p*** **-Value**
CNIO index	0.39	0.15 to 0.99	0.048
SPPB at baseline, points	1.61	1.10 to 2.36	0.015
Age, years	0.99	0.92 to 1.06	0.749
Male sex	0.49	0.17 to 1.42	0.188
BMI, kg/m^2^	1.14	0.94 to 1.37	0.179
COPD	1.01	0.24 to 4.25	0.984
ILD	0.37	0.08 to 1.68	0.196

(**A**) values are presented as unstandardized β coefficients with 95% confidence intervals from the linear ANCOVA model. (**B**) values are presented as proportional odds ratios with 95% confidence intervals from the ordinal logistic regression model. Reference categories were female sex and bronchiectasis for diagnosis. For CNIO, estimates represent the association per 1-SD increase in the standardized CNIO index. The SPPB ordinal logistic regression model was exploratory and should be interpreted cautiously. ANCOVA = analysis of covariance; CI = confidence interval; CNIO = cardio-nutrition-inflammation-oxygen; BMI = body mass index; COPD = chronic obstructive pulmonary disease; ILD = interstitial lung disease; 6MWT = 6 min walk test; SPPB = Short Physical Performance Battery. Model adjusted R^2^ = 0.509 for the 6MWT ANCOVA.

## Data Availability

The data that support the findings of this study are not publicly available because they contain information that could compromise patient privacy, but are available from the corresponding author on reasonable request and with permission of the institutional review board.

## Supplementary Materials

The following supporting information can be downloaded at: https://www.mdpi.com/article/10.3390/nu18121879/s1, Table S1: Sensitivity analyses for SPPB at 3 months; Table S2: Component models for 6MWT and SPPB at 3 months; Table S3: Sensitivity analysis adding FEV1 (% predicted) for 6MWT at 3 months; Table S4: Leave-one-diagnosis-out sensitivity analyses for the CNIO index; Table S5: Correlation matrix of the four CNIO components.

## References

[1] Rochester C.L., Alison J.A., Carlin B., Jenkins A.R., Cox N.S., Bauldoff G., Bhatt S.P., Bourbeau J., Burtin C., Camp P.G. (2023). Pulmonary rehabilitation for adults with chronic respiratory disease: An official american thoracic society clinical practice guideline. Am. J. Respir. Crit. Care Med..

[2] Spruit M.A., Singh S.J., Garvey C., ZuWallack R., Nici L., Rochester C., Hill K., Holland A.E., Lareau S.C., Man W.D. (2013). An official american thoracic society/european respiratory society statement: Key concepts and advances in pulmonary rehabilitation. Am. J. Respir. Crit. Care Med..

[3] McCarthy B., Casey D., Devane D., Murphy K., Murphy E., Lacasse Y. (2015). Pulmonary rehabilitation for chronic obstructive pulmonary disease. Cochrane Database Syst. Rev..

[4] Spruit M.A., Augustin I.M., Vanfleteren L.E., Janssen D.J., Gaffron S., Pennings H.J., Smeenk F., Pieters W., van den Bergh J.J., Michels A.J. (2015). Differential response to pulmonary rehabilitation in copd: Multidimensional profiling. Eur. Respir. J..

[5] Stoffels A.A., De Brandt J., Meys R., van Hees H.W., Vaes A.W., Klijn P., Burtin C., Franssen F.M., van den Borst B., Sillen M.J. (2021). Short physical performance battery: Response to pulmonary rehabilitation and minimal important difference estimates in patients with chronic obstructive pulmonary disease. Arch. Phys. Med. Rehabil..

[6] Crapo R.O., Casaburi R., Coates A.L., Enright P.L., MacIntyre N.R., McKay R.T., Johnson D., Wanger J.S., Zeballos J.R., Bittner V. (2002). Ats statement: Guidelines for the six-minute walk test. Am. J. Respir. Crit. Care Med..

[7] Holland A.E., Spruit M.A., Troosters T., Puhan M.A., Pepin V., Saey D., McCormack M.C., Carlin B.W., Sciurba F.C., Pitta F. (2014). An official european respiratory society/american thoracic society technical standard: Field walking tests in chronic respiratory disease. Eur. Respir. J..

[8] Guralnik J.M., Simonsick E.M., Ferrucci L., Glynn R.J., Berkman L.F., Blazer D.G., Scherr P.A., Wallace R.B. (1994). A short physical performance battery assessing lower extremity function: Association with self-reported disability and prediction of mortality and nursing home admission. J. Gerontol..

[9] Bernabeu-Mora R., Medina-Mirapeix F., Llamazares-Herrán E., García-Guillamón G., Giménez-Giménez L.M., Sánchez-Nieto J.M. (2015). The short physical performance battery is a discriminative tool for identifying patients with copd at risk of disability. Int. J. Chron. Obstr. Pulmon Dis..

[10] Larsson P., Borge C.R., Nygren-Bonnier M., Lerdal A., Edvardsen A. (2018). An evaluation of the short physical performance battery following pulmonary rehabilitation in patients with chronic obstructive pulmonary disease. BMC Res. Notes.

[11] Spruit M.A., Watkins M.L., Edwards L.D., Vestbo J., Calverley P.M., Pinto-Plata V., Celli B.R., Tal-Singer R., Wouters E.F. (2010). Determinants of poor 6-min walking distance in patients with copd: The eclipse cohort. Respir. Med..

[12] Barnes P.J., Celli B.R. (2009). Systemic manifestations and comorbidities of copd. Eur. Respir. J..

[13] Agustí A.G., Noguera A., Sauleda J., Sala E., Pons J., Busquets X. (2003). Systemic effects of chronic obstructive pulmonary disease. Eur. Respir. J..

[14] Nagueh S.F., Smiseth O.A., Appleton C.P., Byrd B.F., Dokainish H., Edvardsen T., Flachskampf F.A., Gillebert T.C., Klein A.L., Lancellotti P. (2016). Recommendations for the evaluation of left ventricular diastolic function by echocardiography: An update from the american society of echocardiography and the european association of cardiovascular imaging. J. Am. Soc. Echocardiogr..

[15] Bouillanne O., Morineau G., Dupont C., Coulombel I., Vincent J.P., Nicolis I., Benazeth S., Cynober L., Aussel C. (2005). Geriatric nutritional risk index: A new index for evaluating at-risk elderly medical patients. Am. J. Clin. Nutr..

[16] Pascual-González Y., López-Sánchez M., Dorca J., Santos S. (2018). Defining the role of neutrophil-to-lymphocyte ratio in copd: A systematic literature review. Int. J. Chron. Obstr. Pulmon Dis..

[17] Jacobs S.S., Krishnan J.A., Lederer D.J., Ghazipura M., Hossain T., Tan A.M., Carlin B., Drummond M.B., Ekström M., Garvey C. (2020). Home oxygen therapy for adults with chronic lung disease. An official american thoracic society clinical practice guideline. Am. J. Respir. Crit. Care Med..

[18] Park J.H., Marwick T.H. (2011). Use and limitations of e/e’ to assess left ventricular filling pressure by echocardiography. J. Cardiovasc. Ultrasound.

[19] Matsumura T., Mitani Y., Oki Y., Fujimoto Y., Ohira M., Kaneko H., Kawashima T., Nishio M., Ishikawa A. (2015). Comparison of geriatric nutritional risk index scores on physical performance among elderly patients with chronic obstructive pulmonary disease. Heart Lung.

[20] Lee H., Um S.J., Kim Y.S., Kim D.K., Jang A.S., Choi H.S., Kim Y.H., Kim T.E., Yoo K.H., Jung K.S. (2016). Association of the neutrophil-to-lymphocyte ratio with lung function and exacerbations in patients with chronic obstructive pulmonary disease. PLoS ONE.

[21] Günay E., Sarınç Ulaşlı S., Akar O., Ahsen A., Günay S., Koyuncu T., Unlü M. (2014). Neutrophil-to-lymphocyte ratio in chronic obstructive pulmonary disease: A retrospective study. Inflammation.

[22] Holland A.E., Hill C.J., Rasekaba T., Lee A., Naughton M.T., McDonald C.F. (2010). Updating the minimal important difference for six-minute walk distance in patients with chronic obstructive pulmonary disease. Arch. Phys. Med. Rehabil..

[23] Puhan M.A., Chandra D., Mosenifar Z., Ries A., Make B., Hansel N.N., Wise R.A., Sciurba F. (2011). The minimal important difference of exercise tests in severe copd. Eur. Respir. J..

[24] Puhan M.A., Mador M.J., Held U., Goldstein R., Guyatt G.H., Schünemann H.J. (2008). Interpretation of treatment changes in 6-minute walk distance in patients with copd. Eur. Respir. J..

[25] Furutate R., Ishii T., Motegi T., Hattori K., Kusunoki Y., Gemma A., Kida K. (2016). The neutrophil to lymphocyte ratio is related to disease severity and exacerbation in patients with chronic obstructive pulmonary disease. Intern. Med..

