# Supplementary material for: A Derivation Study of a Cardio-Nutrition-Inflammation-Oxygen Index and 3-Month Functional Outcomes After Outpatient Pulmonary Rehabilitation
Source: Nutrients. 2026 Jun 11;18(12):1879. doi: 10.3390/nu18121879 (PMC13305439; doi:10.3390/nu18121879)
Supplement: Supplementary file 1 [file nutrients-18-01879-s001.zip › nutrients-4351403-supplementary.pdf]

Supplementary Table S1. Sensitivity Analyses for SPPB at 3 Months

Panel A. Linear ANCOVA Sensitivity Analysis for SPPB at 3 Months

**Variable**	**β**	**95% CI**	**p-value**
CNIO index	-1.07	-2.11 to -0.02	0.045
SPPB at baseline, points	0.45	0.02 to 0.89	0.040
Age, years	-0.01	-0.09 to 0.07	0.843
Male sex	-0.94	-2.18 to 0.30	0.136
BMI, kg/m²	0.15	-0.08 to 0.37	0.194
COPD	0.21	-1.53 to 1.94	0.812
ILD	-0.96	-2.70 to 0.78	0.272

Panel B. Ordinal Logistic Regression for SPPB at 3 Months Additionally Adjusted for FEV1 (% Predicted)

**Variable**	**OR**	**95% CI**	**p-value**
CNIO index	0.39	0.15 to 0.99	0.047
SPPB at baseline, points	1.60	1.09 to 2.35	0.017
Age, years	0.99	0.92 to 1.06	0.743
Male sex	0.44	0.14 to 1.41	0.166
BMI, kg/m²	1.12	0.93 to 1.36	0.238
COPD	0.91	0.20 to 4.12	0.900
ILD	0.37	0.08 to 1.69	0.199
FEV1 (% predicted)	1.01	0.96 to 1.07	0.641

Panel A values are presented as unstandardized β coefficients with 95% confidence intervals from the linear ANCOVA model. Panel B values are presented as proportional odds ratios with 95% confidence intervals from the ordinal logistic regression model. For CNIO, estimates represent the association per 1-SD increase in the standardized CNIO index. Reference categories were female sex and bronchiectasis for diagnosis. ANCOVA = analysis of covariance; CI = confidence interval; CNIO = cardio-nutrition-inflammation-oxygen; OR = odds ratio; SPPB = Short Physical Performance Battery; FEV1 = forced expiratory volume in 1 second; BMI = body mass index; COPD = chronic obstructive pulmonary disease; ILD = interstitial lung disease. Model adjusted $R^2 = 0.437$ for the linear ANCOVA model.

Supplementary Table S2. Component Models for 6MWT and SPPB at 3 Months

Panel A. 6MWT at 3 Months

Variable	β	95% CI	p-value
E/e′	-6.87	-17.21 to 3.48	0.188
GNRI	5.94	2.07 to 9.82	0.003
ln(NLR)	-12.99	-55.15 to 29.18	0.539
Resting oxygen requirement, L/min	-10.75	-36.79 to 15.29	0.411
6MWT at baseline, m	0.45	0.06 to 0.84	0.025
Age, years	0.01	-2.84 to 2.87	0.994
Male sex	-1.68	-45.43 to 42.08	0.939
BMI, kg/m²	-0.88	-8.78 to 7.02	0.824
COPD	-23.61	-82.22 to 34.99	0.422
ILD	-73.42	-133.72 to -13.13	0.018

Panel B. SPPB at 3 Months

Variable	β	95% CI	p-value
E/e′	-0.08	-0.39 to 0.24	0.632
GNRI	0.03	-0.09 to 0.15	0.647
ln(NLR)	-1.25	-2.59 to 0.09	0.066
Resting oxygen requirement, L/min	-0.60	-1.42 to 0.22	0.149
SPPB at baseline, points	0.47	0.02 to 0.91	0.041
Age, years	-0.02	-0.11 to 0.06	0.578
Male sex	-0.94	-2.21 to 0.34	0.145
BMI, kg/m²	0.18	-0.06 to 0.42	0.135
COPD	0.29	-1.56 to 2.14	0.753
ILD	-0.92	-2.76 to 0.92	0.321

Values are presented as unstandardized β coefficients with 95% confidence intervals from exploratory linear ANCOVA component models. Reference categories were female sex and bronchiectasis for diagnosis. These component models were exploratory and should be interpreted cautiously because multiple related CNIO components and clinical covariates were entered simultaneously in a modest sample. ANCOVA = analysis of covariance; CI = confidence interval; GNRI = Geriatric Nutritional Risk Index; ln(NLR) = natural log-transformed neutrophil-to-lymphocyte ratio; BMI = body mass index; COPD = chronic obstructive pulmonary disease; ILD = interstitial lung disease; 6MWT = 6-minute walk test; SPPB = Short Physical Performance Battery. Model adjusted R^2 = 0.519 for 6MWT and 0.415 for SPPB.

Supplementary Table S3. Sensitivity Analysis Adding FEV1 (% Predicted) for 6MWT at 3 Months

Variable	β	95% CI	p-value
CNIO index	-43.46	-78.07 to -8.86	0.015
6MWT at baseline, m	0.48	0.10 to 0.87	0.014
Age, years	-0.66	-3.34 to 2.03	0.625
Male sex	-0.79	-48.03 to 46.45	0.973
BMI, kg/m²	1.41	-6.31 to 9.13	0.715
COPD	-19.36	-78.50 to 39.78	0.514
ILD	-62.18	-120.43 to -3.93	0.037
FEV1 (% predicted)	-0.03	-2.19 to 2.13	0.978

Values are presented as unstandardized β coefficients with 95% confidence intervals from the FEV1-adjusted linear ANCOVA model. Reference categories were female sex and bronchiectasis for diagnosis. For CNIO, β represents the change in 3-month 6MWT distance per 1-SD increase in the standardized CNIO index. ANCOVA = analysis of covariance; CI = confidence interval; CNIO = cardio-nutrition-inflammation-oxygen; FEV1 = forced expiratory volume in 1 second; BMI = body mass index; COPD = chronic obstructive pulmonary disease; ILD = interstitial lung disease; 6MWT = 6-minute walk test. Model adjusted $R^2 = 0.499$.

Supplementary Table S4. Leave-One-Diagnosis-Out Sensitivity Analyses for the CNIO Index

Subset	Outcome	N	Measure	Estimate for CNIO	95% CI	p-value
Excluding COPD	6MWT at 3 months	35	β	-65.64	-104.07 to -27.21	0.002
Excluding COPD	SPPB at 3 months	35	OR	0.33	0.13 to 0.84	0.020
Excluding ILD	6MWT at 3 months	40	β	-34.08	-74.58 to 6.43	0.096
Excluding ILD	SPPB at 3 months	40	OR	0.65	0.24 to 1.79	0.408
Excluding bronchiectasis	6MWT at 3 months	45	β	-61.10	-83.57 to -38.63	<0.001
Excluding bronchiectasis	SPPB at 3 months	45	OR	0.19	0.08 to 0.45	<0.001

Reduced models were adjusted for the baseline value of the corresponding outcome, age, sex, and BMI. Diagnosis category was omitted by design in the leave-one-diagnosis-out analyses. For 6MWT, estimates are β coefficients in meters per 1-SD increase in the standardized CNIO index from linear ANCOVA models. For SPPB, estimates are proportional odds ratios for being in a higher 3-month SPPB category per 1-SD increase in the standardized CNIO index from ordinal logistic regression models. CNIO = cardio-nutrition-inflammation-oxygen; CI = confidence interval; BMI = body mass index; COPD = chronic obstructive pulmonary disease; ILD = interstitial lung disease; 6MWT = 6-minute walk test; SPPB = Short Physical Performance Battery. These leave-one-diagnosis-out analyses were exploratory pattern-consistency analyses.

Supplementary Table S5. Correlation Matrix of the Four CNIO Components

Variable	E/e′	GNRI	ln(NLR)	Resting oxygen requirement
E/e′	1.00	-0.26	0.27	0.29
GNRI	-0.26	1.00	-0.19	-0.12
ln(NLR)	0.27	-0.19	1.00	0.06
Resting oxygen requirement	0.29	-0.12	0.06	1.00

Values are Pearson correlation coefficients. CNIO = cardio-nutrition-inflammation-oxygen; GNRI = Geriatric Nutritional Risk Index; ln(NLR) = natural log-transformed neutrophil-to-lymphocyte ratio.
